# Exposomics: From Daily-Life Exposure Monitoring to Precision Prevention

**Published:** 2026-01-16

**Authors:** Aristides Tsatsakis, Dimosthenis A. Sarigiannis, Michael Aschner, Ramin Rezaee

**Affiliations:** 1 Center of Toxicology Science & Research, Division of Morphology, Medical School, University of Crete, Voutes Campus, 71003 Heraklion, Greece; 2 Faculty of Health Sciences and Human Development, Universidad ECOTEC, Samborondón 13.5 Km, Samborondón EC092302, Ecuador; 3 I. M. Sechenov First Moscow State Medical University, Moscow 119435, Russia; 4 National Hellenic Research Foundation, 48 Vassileos Constantinou Avenue, 11635 Athens, Greece; 5 Environmental Engineering Laboratory, Department of Chemical Engineering, Aristotle University of Thessaloniki, University Campus, 54124 Thessaloniki, Greece; 6 HERACLES Research Center on the Exposome and Health, Center for Interdisciplinary Research and Innovation, Balkan Center, 57001 Thessaloniki, Greece; 7 Research Center on Complex Risk and Data Analysis, University School for Advanced Studies IUSS, 27100 Pavia, Italy; 8 Department of Molecular Pharmacology, Albert Einstein College of Medicine, Bronx, NY 10461, USA; 9 Applied Biomedical Research Center, Basic Sciences Research Institute, Mashhad University of Medical Sciences, Mashhad 91778-99191, Iran

As founding editors, we are pleased to announce the launch of the *International Journal of Exposome and Toxicology* (*IJET*), a dedicated forum for research at the interface of exposure science, exposomics and mechanistic toxicology, with a clear focus on real-life exposure scenarios.

The exposome was introduced to capture the totality of environmental, occupational, lifestyle and social exposures across the life course, starting from preconception and continuing throughout life. In our work, we have advocated for an operational view of the exposome as a time-resolved “expotype”—the vector of external and internal exposures that, together with the genotype, shapes the phenotype and ultimately the risk of disease, and can serve as a practical tool for improved health risk assessment [1]. This perspective reframes the classic “nature versus nurture” debate into a systems paradigm of dynamic interactions between environmental exposures, endogenous processes and genetic expression [2,3]. Twin studies and exposure science also support the central role of environmental determinants in health outcomes [4,5].

Exposomics provides the methodological toolbox to characterise both the external and the internal exposome. Beyond environmental monitoring, it integrates human biomonitoring, refined exposure assessment (multi-route/multi-pathway), internal dosimetry and physiologically based biokinetic (PBPK/PBTK) modelling with multi-omics profiling (e.g., metabolomics, transcriptomics, epigenomics and proteomics) and exposome-wide association studies (ExWAS). When these data streams are analysed jointly, they can reduce exposure misclassification, strengthen causal inference and help translate exposure–response relationships into targeted prevention strategies and policy-relevant risk management [6]. The HERACLES project illustrates this approach by combining environmental measurements, biomonitoring, omics and clinical endpoints to investigate child neurodevelopment in communities affected by chronic pollution while accounting for socioeconomic and lifestyle modifiers [2,3].

Among the integrative biomarkers relevant to the internal exposome, telomere length and dynamics are increasingly explored as indicators of cumulative biological stress and ageing. Changes in telomere biology may reflect the combined imprint of chronic low-dose exposures acting through oxidative stress, inflammation, mitochondrial dysfunction and epigenetic regulation, and may therefore support exposome-based risk stratification [7].

In real life, humans are exposed daily to complex mixtures of chemicals and non-chemical stressors through air, water, diet and the built environment. Yet traditional risk assessment still often relies on single-chemical paradigms and high-dose testing, potentially overlooking mixture effects (additive, antagonistic, synergistic or supra-additive) and susceptible subgroups [8,9]. The Real-Life Risk Simulation (RLRS) approach has been proposed to bridge this gap by aligning exposure scenarios, mechanistic evidence and modelling to better support cumulative risk assessment and decision-making [6,10].

Progress in this field depends on integration: combining high-quality epidemiology with innovative experimental systems, new approach methodologies and in silico tools, while explicitly linking external exposure metrics to biologically effective doses and toxicity pathway perturbations. Such evidence integration is essential for moving from associations to causality and for ensuring that exposome science meaningfully informs regulation and public health action [6,11].

The *IJET* is established as a gold open access, peer-reviewed journal with the mission of advancing evidence-based toxicological research, with particular emphasis on innovations in single and multiple exposure assessment. By providing an international platform for interdisciplinary scholarship, *IJET* seeks to elucidate the complex relationships between environmental exposures and biological and health outcomes across the life course. The journal welcomes high-quality original research and authoritative reviews spanning preclinical (*in silico*, *in vitro*, and in *vivo*) as well as clinical investigations in the following areas:

- Biomonitoring and exposure assessment
  Research using cutting-edge approaches for biomonitoring and exposure science, including targeted and untargeted measurements, high-resolution mass spectrometry, sensors and wearables, geospatial analytics, and human biomonitoring to characterise external exposures and translate them into internal exposure metrics and biologically effective doses.
- Exposome and ecosystem-health links
  Innovative research linking ecosystem integrity, environmental contamination and human health within One Health/planetary health perspectives, including studies that connect changes in environmental media to the human exposome and health impacts across the life course.
- Policy and regulation
  Studies aimed at informing policy-making and regulation through exposome-informed evidence, cumulative and mixture risk assessment, and translation of mechanistic and epidemiological findings into decision-relevant metrics.
- Epidemiological, clinical and experimental toxicology
  Epidemiological, clinical and experimental (*in vivo*, *in vitro* and *in silico*) studies investigating adverse effects of natural and synthetic toxicants, including exposome-wide association designs, vulnerable populations and critical windows of susceptibility.
- Protective and mitigating effects
  Human, animal and cellular studies reporting protective or mitigating effects (e.g., nutritional, behavioural, pharmacological or technological interventions) against toxic insults, and research on resilience factors that modify exposure–effect relationships.
- Analytical evaluations and risk assessment
  Analytical studies and risk assessments of human exposure to chemicals via different routes (food, environmental and occupational exposure, pharmaceuticals and consumer products), incorporating internal dosimetry, PBPK/PBTK modelling, real-life risk simulation and explicit treatment of uncertainty and variability.
- Exposure reduction and mitigation strategies
  Research on the development, evaluation and implementation of strategies to reduce or mitigate exposure to toxic agents at individual, community and population levels, including precision prevention approaches.
- Novel toxicity assessment methods
  Studies describing the development, validation or application of innovative toxicity assessment methods and testing strategies, including new approach methodologies (NAMs), high-throughput screening and advanced in vitro models (e.g., organoids and organ-on-chip platforms).
- Mechanistic toxicology
  Research aimed at unravelling molecular and cellular mechanisms underlying adverse health impacts of exposures, including pathway perturbations, adverse outcome pathways (AOPs) and systems toxicology approaches.
- Chemicals of emerging concern
  Investigations into the toxicological properties, exposure patterns, mixture behaviour and health implications of chemicals of emerging concern (e.g., PFAS, microplastics, nanomaterials and novel consumer-product ingredients).

We strongly encourage authors to provide a clear indication of the real-life relevance of the studied exposure(s): typical concentration ranges, exposure routes, mixture context, and (where possible) biologically effective dose metrics. We also welcome submissions that explicitly discuss implications for risk assessment, regulation and precision prevention in public health.
